# Advantages of negative pressure wound therapy with instillation of super oxidized solution and dwell time in diabetic foot syndrome: a rare case report

**DOI:** 10.1093/jscr/rjab167

**Published:** 2021-05-18

**Authors:** Bassey Enodien, Dana Hendie, Gioia Pozza, Alexei Lyzikov, Stephanie Taha-Mehlitz, Anas Taha

**Affiliations:** Department of Surgery, Wetzikon Hospital, Wetzikon, CHE, Switzerland; Department of Surgery, College of Medicine, Sulaiman Al Rajhi University, Albukairyah, Saudi Arabia; Department of Surgery, Azienda Sanitaria Universitaria Integrata di Trieste, Friuli-Venezia Giulia, Trieste, Italy; Department of Cardiovaskular Surgery, Gomel State Medical University, Gomel, BY, Belarus; Department of Visceral Surgery, Clarunis University Center for Gastrointestinal and Liver Diseases, St. Clara Hospital and University Hospital, Basel, CHE, Switzerland; Department of Visceral and Thoracic Surgery, Cantonal Hospital Winterthur, Winterthur, CHE, Switzerland

## Abstract

Negative pressure wound therapy (NPWT) with instillation therapy (V.A.C. Vera-Flow™) and dwell time (NPWTi-d) is an innovative method for complex wound healing. NPWTi-d combines vacuum-aided drainage of wounds with the precise distribution of topical cleansing solution over the wounds. Furthermore, super oxidized solutions have illustrated their ability to potentiate wound healing and decrease bacterial contamination. Furthermore, aided with this method, infected wounds can be disinfected. If surgical debridement or removal of the infected site is not possible or desired. Therefore, in the case of a 66-year-old patient with diabetic foot syndrome (DFS) with severe infection, our approach was to couple NPWTi-d with instillation and dwelling of super oxidized solution to bolster benefits. This is the first case report using NPWTi-d with instillation of super oxidized solution in DFS in Switzerland. This case indicates that this approach is beneficial in the treatment of complex and critically infected wounds in DFS.

## INTRODUCTION

Diabetic foot syndrome (DFS) is one of the main causes of morbidity and mortality in diabetic patients. It is defined by the World Health Organization as an ‘ulceration of the foot (distally from the ankle and including the ankle) associated with neuropathy and different grades of ischemia and infection’ [1]. Many reports and reviews concerning DFS have demonstrated that management with early diagnostics is crucial in the treatment of DFS [2]. Therefore, an increasing amount of management approaches have been proposed.

In this case, after surgical debridement, we combined the advantages of the negative pressure wound therapy with instillation and dwell time (NPWTi-d) and the advantages of instillation of super oxidized solution.

## CASE PRESENTATION

**Figure 1:**
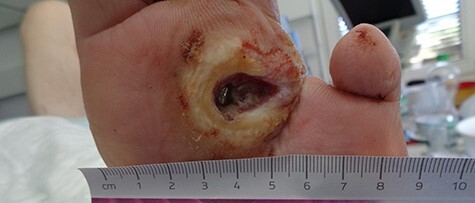
Infected ulcer and osteomyelitis of metatarsal bone II (probe to bone positive).

A 66-year-old male, with a case of complicated type 2 diabetes mellitus, peripheral artery occlusive disease and left diabetic foot with prior trans-metatarsal amputation of dig. III and IV presented with increasingly infected ulcers and osteomyelitis of dig. II and metatarsal II (Fig. 1) after an episode of severe gastroenteritis with diarrhea.

**Figure 2:**
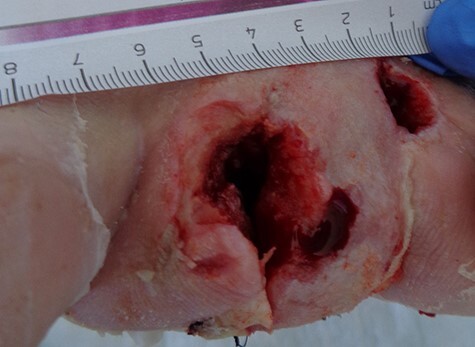
After admission a trans-metatarsal amputation of dig. II was conducted, wound was left open.

After admission, a trans-metatarsal amputation of dig. II was conducted and the wound was left open with wet dressing (Fig. 2). The following day the situation was evaluated. Due to the severe infection of the wound, the recommendation to amputate the forefoot was given. Considering the patient’s will to keep the remaining forefoot and biomechanical considerations, an attempt was made to retain it.

After surgical debridement, NPWTi-d with super oxidized solution was instilled, ActiMaris sensitive™. It is an alkaline (ph 8.5–10) hypertonic sea salt solution (NaCl 1.2% and NaOCl 0.04%).

**Figure 3:**
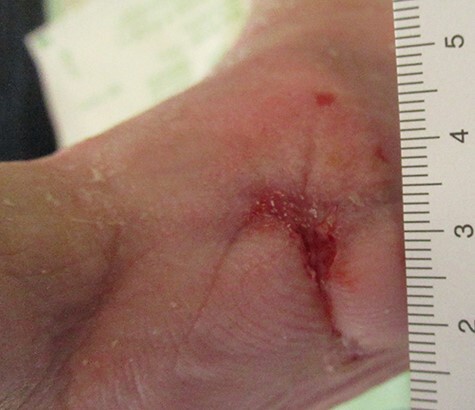
Wound 6 weeks after trans-metatarsal amputation of dig. II.

A current review detailing the clinical application of NPWTi-d showed mean dwell times of 14.23 min (95% confidence interval [CI]: 10.88–17.59) and instillation cycles every 4.17 ± 2.32 hourly were appropriate [3], even though commonly normal saline was used for instillation [3]. Using super oxidized solution, we set dwelling time to 20 min every 6 h [4]. Pressure was set to negative −125 mmHg and dressing changes were performed every 2–3 days [4]. The spread of infection was prevented with the combination of antibiotics. After 14 days of treatment, NPWTi-d with super oxidized solution was stopped because of rapid recovery of the wound and growth of granulation tissue. A negative pressure wound therapy without installation (NPWT) was extended for another 4 weeks until wound closure was attained (Fig. 3). The treatment lasted for 6 weeks until wound closure was achieved.

## DISCUSSION

DFS is caused by a combination of vascular diseases, neuropathy and deprived wound healing. These conditions facilitate the emergence of foot ulcers and infections. Earlier publications have established that diabetic foot ulcers have an annual incidence of 1–4% and a prevalence of 5–10% [2]. Patients with DFS have a higher risk of amputation and death, and thus optimal treatment to limit the rate of amputations is vital.

NPWT is a vacuum-aided drainage of wounds using a suction pump, dressings and tubing to promote the healing of wounds [5]. NPWTi-d is a system with an instillation feature and specialized dressings that allows the super-oxidized solutions to dwell in the wound to prolong contact and cleanse the wound bed. The super oxidized solutions would then bolster wound healing and decrease bacterial contamination [6].

Earlier publications detailed the advantages of NPWT overall [7], NPWTi-d in patients with deep tissue infection [7] as well as NPWTi-d in patients suffering from DFS [8]. NPWTi-d has been shown to be superior to NPWT in the treatment of severely infected wounds [9]. Several published reviews and recommendations exist for the use of NPWTi-d in both acute and chronic wounds [9, 10]. Furthermore, other journals elucidated the advantages of super oxidized solution [11] in the management of DFS reducing reinfection rates [12] and obviating the necessity for debridement procedures [12].

In this case, the patient presented with a complicated case of type 2 diabetes mellites, peripheral artery occlusive disease, left diabetic foot with prior trans-metatarsal amputation of dig. III/IV and with increasingly infected ulcers and Osteomyelitis of dig. II and metatarsal II. The comorbidities and overall condition had compromised wound healing. NPWTi-d and the advantages of instillation with super oxidized solution [8, 11] were used to cleanse the wound surface, provide a protective barrier and enable the progression of wound healing.

Historically, these wounds have been managed with daily wet-to-dry dressing changes with sterile gauze. This is especially worrisome given the challenging anatomical topography of the wound, the septic state of the wound and the vulnerable neighboring bone structures. Cleanliness of the wound should be realized as soon as possible. Therefore, NPWT, as well as NPWTi-d, have been gaining prominence in the treatment of patients with complex comorbidities [7, 9, 10, 12]. In patients with diabetic foot syndrome specifically, every advantage needs to be utilized to boost the regression of infection and progression of wound healing.

## CONCLUSIONS

In our clinical practice, NPWTi-d with instillation of super oxidized solution helped to promote wound healing, to remove infectious material, and to prevent the infection from spreading in combination with antibiotics. This method may provide benefit even in patients with DFS, comorbidities and largely infected wounds.
